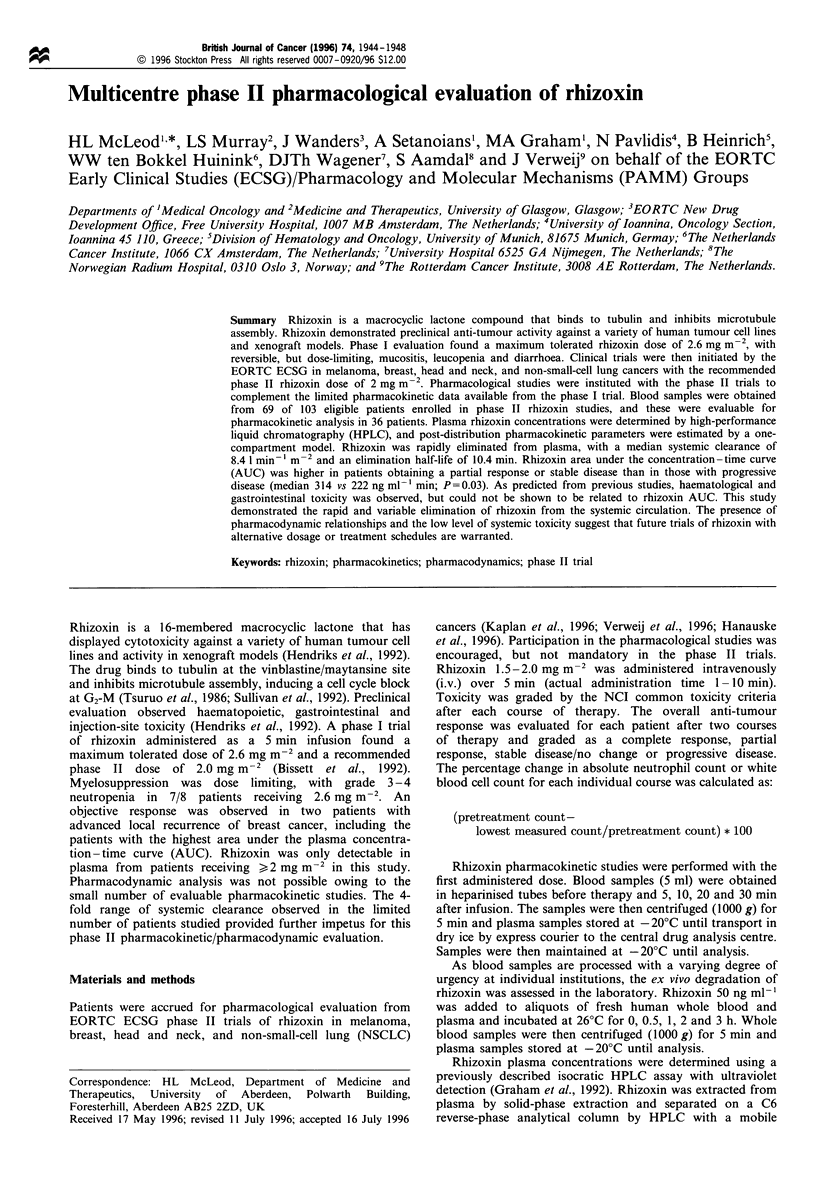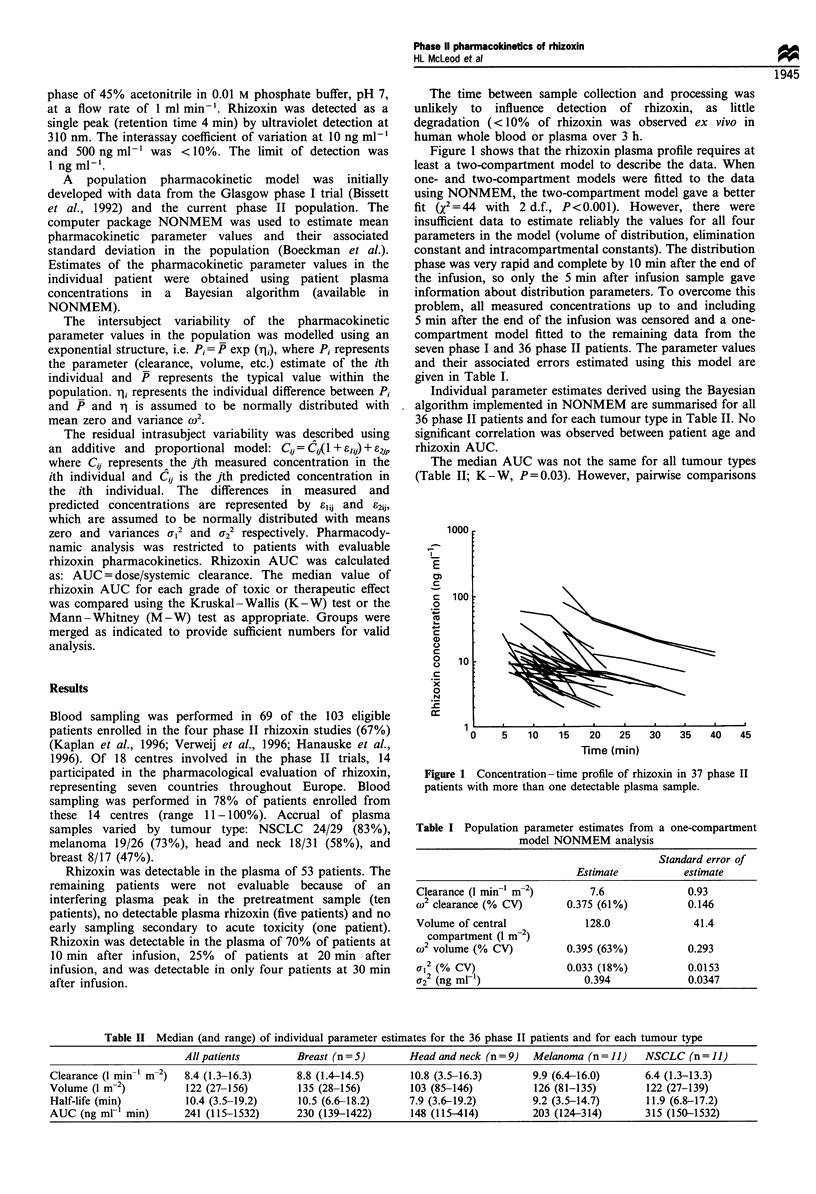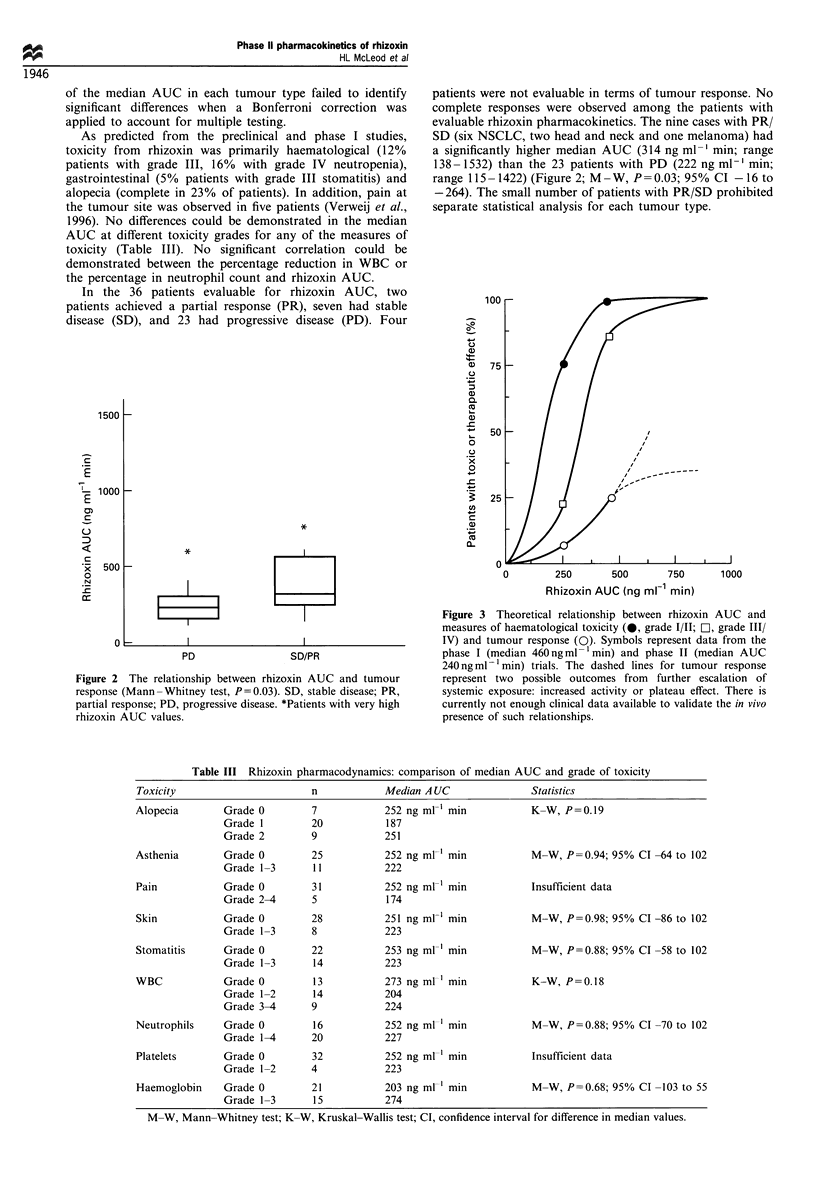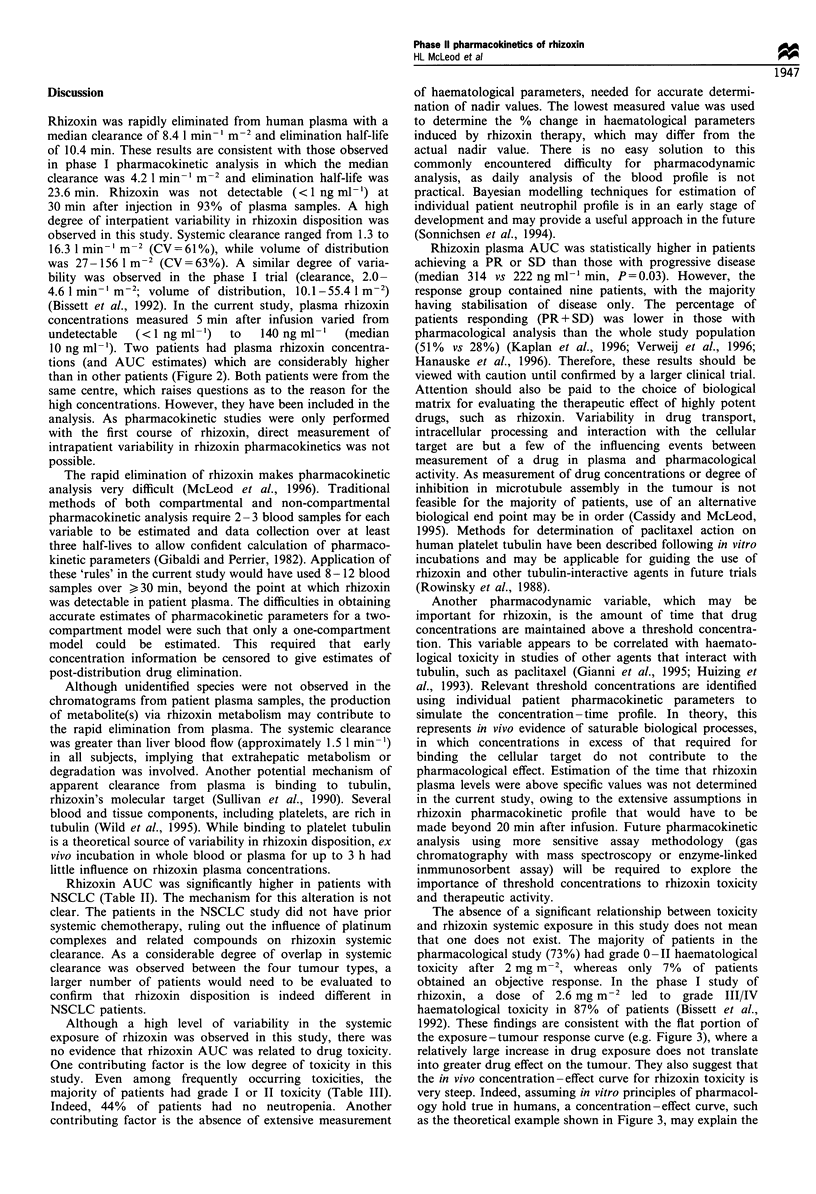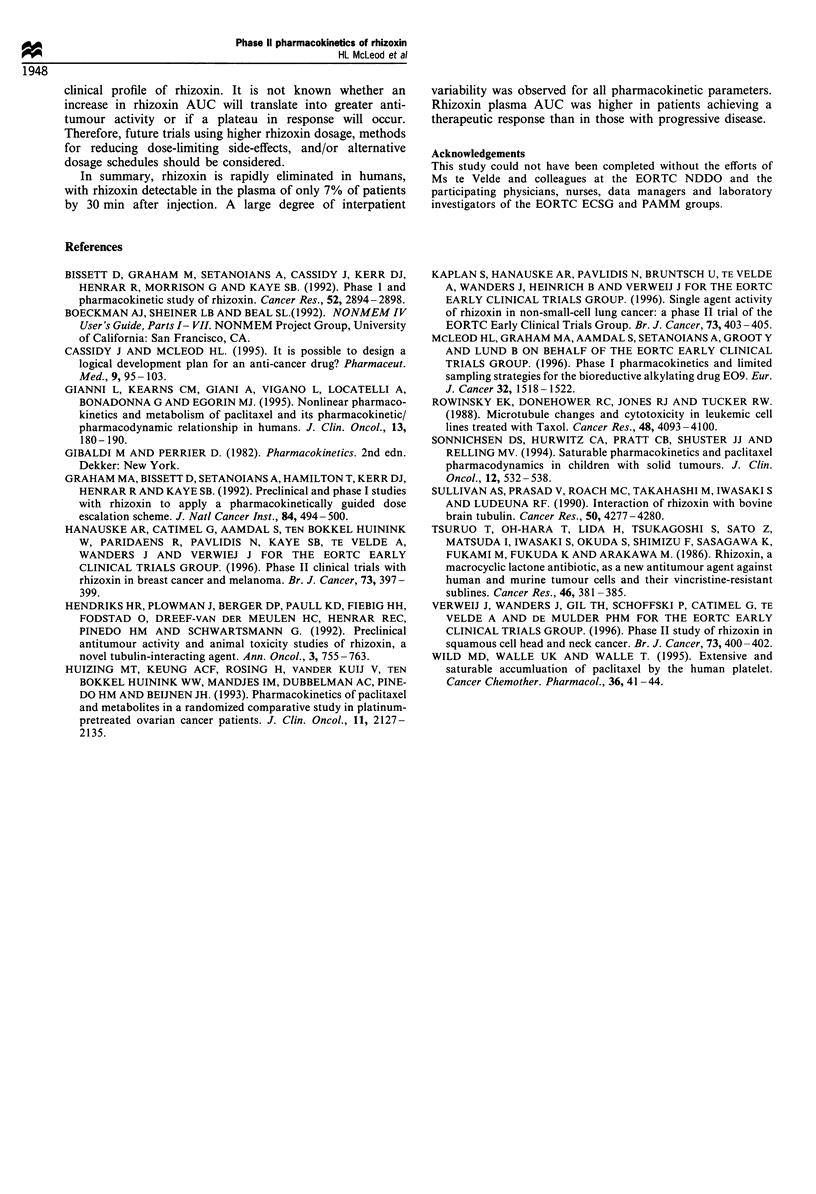# Multicentre phase II pharmacological evaluation of rhizoxin. Eortc early clinical studies (ECSG)/pharmacology and molecular mechanisms (PAMM) groups.

**DOI:** 10.1038/bjc.1996.657

**Published:** 1996-12

**Authors:** H. L. McLeod, L. S. Murray, J. Wanders, A. Setanoians, M. A. Graham, N. Pavlidis, B. Heinrich, W. W. ten Bokkel Huinink, D. J. Wagener, S. Aamdal, J. Verweij

**Affiliations:** Department of Medical Oncology, University of Glasgow, UK.

## Abstract

Rhizoxin is a macrocyclic lactone compound that binds to tubulin and inhibits microtubule assembly. Rhizoxin demonstrated preclinical anti-tumour activity against a variety of human tumour cell lines and xenograft models. Phase I evaluation found a maximum tolerated rhizoxin dose of 2.6 mg m-2, with reversible, but dose-limiting, mucositis, leucopenia and diarrhoea. Clinical trials were then initiated by the EORTC ECSG in melanoma, breast, head and neck, and non-small-cell lung cancers with the recommended phase II rhizoxin dose of 2 mg m-2. Pharmacological studies were instituted with the phase II trials to complement the limited pharmacokinetic data available from the phase I trial. Blood samples were obtained from 69 of 103 eligible patients enrolled in phase II rhizoxin studies, and these were evaluable for pharmacokinetic analysis in 36 patients. Plasma rhizoxin concentrations were determined by high-performance liquid chromatography (HPLC), and post-distribution pharmacokinetic parameters were estimated by a one-compartment model. Rhizoxin was rapidly eliminated from plasma, with a median systemic clearance of 8.41 min-1 m-2 and an elimination half-life of 10.4 min. Rhizoxin area under the concentration-time curve (AUC) was higher in patients obtaining a partial response or stable disease than in those with progressive disease (median 314 vs 222 ng ml-1 min; P = 0.03). As predicted from previous studies, haematological and gastrointestinal toxicity was observed, but could not be shown to be related to rhizoxin AUC. This study demonstrated the rapid and variable elimination of rhizoxin from the systemic circulation. The presence of pharmacodynamic relationships and the low level of systemic toxicity suggest that future trials of rhizoxin with alternative dosage or treatment schedules are warranted.


British Journal of Cancer (1996) 74, 1944-1948
© 1996 Stockton Press All rights reserved 0007-0920/96 $12.00

### Multicentre phase II pharmacological evaluation of rhizoxin

HL McLeod[1,*], LS Murray[2], J Wanders[3], A Setanoians[1], MA Graham[1], N Pavlidis[4], B Heinrich[5], WW ten Bokkel Huinink[6], DJTh Wagener[7], S Aamdal[8] and J Verweij[9] on behalf of the EORTC Early Clinical Studies (ECSG)/Pharmacology and Molecular Mechanisms (PAMM) Groups

*Departments of [1]Medical Oncology and [2]Medicine and Therapeutics, University of Glasgow, Glasgow; [3]EORTC New Drug Development Office, Free University Hospital, 1007 MB Amsterdam, The Netherlands; [4]University of Ioannina, Oncology Section, Ioannina 45 110, Greece; [5]Division of Hematology and Oncology, University of Munich, 81675 Munich, Germay; [6]The Netherlands Cancer Institute, 1066 CX Amsterdam, The Netherlands; [7]University Hospital 6525 GA Nijmegen, The Netherlands; [8]The Norwegian Radium Hospital, 0310 Oslo 3, Norway; and [9]The Rotterdam Cancer Institute, 3008 AE Rotterdam, The Netherlands.*

**Summary** Rhizoxin is a macrocyclic lactone compound that binds to tubulin and inhibits microtubule assembly. Rhizoxin demonstrated preclinical anti-tumour activity against a variety of human tumour cell lines and xenograft models. Phase I evaluation found a maximum tolerated rhizoxin dose of 2.6 mg $m^{-2}$, with reversible, but dose-limiting, mucositis, leucopenia and diarrhoea. Clinical trials were then initiated by the EORTC ECSG in melanoma, breast, head and neck, and non-small-cell lung cancers with the recommended phase II rhizoxin dose of 2 mg $m^{-2}$. Pharmacological studies were instituted with the phase II trials to complement the limited pharmacokinetic data available from the phase I trial. Blood samples were obtained from 69 of 103 eligible patients enrolled in phase II rhizoxin studies, and these were evaluable for pharmacokinetic analysis in 36 patients. Plasma rhizoxin concentrations were determined by high-performance liquid chromatography (HPLC), and post-distribution pharmacokinetic parameters were estimated by a one-compartment model. Rhizoxin was rapidly eliminated from plasma, with a median systemic clearance of 8.4 l $min^{-1}$ $m^{-2}$ and an elimination half-life of 10.4 min. Rhizoxin area under the concentration–time curve (AUC) was higher in patients obtaining a partial response or stable disease than in those with progressive disease (median 314 *vs* 222 ng $ml^{-1}$ min; $P=0.03$). As predicted from previous studies, haematological and gastrointestinal toxicity was observed, but could not be shown to be related to rhizoxin AUC. This study demonstrated the rapid and variable elimination of rhizoxin from the systemic circulation. The presence of pharmacodynamic relationships and the low level of systemic toxicity suggest that future trials of rhizoxin with alternative dosage or treatment schedules are warranted.

**Keywords:** rhizoxin; pharmacokinetics; pharmacodynamics; phase II trial

Rhizoxin is a 16-membered macrocyclic lactone that has displayed cytotoxicity against a variety of human tumour cell lines and activity in xenograft models (Hendriks *et al.*, 1992). The drug binds to tubulin at the vinblastine/maytansine site and inhibits microtubule assembly, inducing a cell cycle block at $G_2$-M (Tsuruo *et al.*, 1986; Sullivan *et al.*, 1992). Preclinical evaluation observed haematopoietic, gastrointestinal and injection-site toxicity (Hendriks *et al.*, 1992). A phase I trial of rhizoxin administered as a 5 min infusion found a maximum tolerated dose of 2.6 mg $m^{-2}$ and a recommended phase II dose of 2.0 mg $m^{-2}$ (Bissett *et al.*, 1992). Myelosuppression was dose limiting, with grade 3–4 neutropenia in 7/8 patients receiving 2.6 mg $m^{-2}$. An objective response was observed in two patients with advanced local recurrence of breast cancer, including the patients with the highest area under the plasma concentration–time curve (AUC). Rhizoxin was only detectable in plasma from patients receiving $\geqslant 2$ mg $m^{-2}$ in this study. Pharmacodynamic analysis was not possible owing to the small number of evaluable pharmacokinetic studies. The 4-fold range of systemic clearance observed in the limited number of patients studied provided further impetus for this phase II pharmacokinetic/pharmacodynamic evaluation.

### Materials and methods

Patients were accrued for pharmacological evaluation from EORTC ECSG phase II trials of rhizoxin in melanoma, breast, head and neck, and non-small-cell lung (NSCLC) cancers (Kaplan *et al.*, 1996; Verweij *et al.*, 1996; Hanauske *et al.*, 1996). Participation in the pharmacological studies was encouraged, but not mandatory in the phase II trials. Rhizoxin 1.5–2.0 mg $m^{-2}$ was administered intravenously (i.v.) over 5 min (actual administration time 1–10 min). Toxicity was graded by the NCI common toxicity criteria after each course of therapy. The overall anti-tumour response was evaluated for each patient after two courses of therapy and graded as a complete response, partial response, stable disease/no change or progressive disease. The percentage change in absolute neutrophil count or white blood cell count for each individual course was calculated as:

(pretreatment count–
lowest measured count/pretreatment count) * 100

Rhizoxin pharmacokinetic studies were performed with the first administered dose. Blood samples (5 ml) were obtained in heparinised tubes before therapy and 5, 10, 20 and 30 min after infusion. The samples were then centrifuged (1000 ***g***) for 5 min and plasma samples stored at −20°C until transport in dry ice by express courier to the central drug analysis centre. Samples were then maintained at −20°C until analysis.

As blood samples are processed with a varying degree of urgency at individual institutions, the *ex vivo* degradation of rhizoxin was assessed in the laboratory. Rhizoxin 50 ng $ml^{-1}$ was added to aliquots of fresh human whole blood and plasma and incubated at 26°C for 0, 0.5, 1, 2 and 3 h. Whole blood samples were then centrifuged (1000 ***g***) for 5 min and plasma samples stored at −20°C until analysis.

Rhizoxin plasma concentrations were determined using a previously described isocratic HPLC assay with ultraviolet detection (Graham *et al.*, 1992). Rhizoxin was extracted from plasma by solid-phase extraction and separated on a C6 reverse-phase analytical column by HPLC with a mobile

Correspondence: HL McLeod, Department of Medicine and Therapeutics, University of Aberdeen, Polwarth Building, Foresterhill, Aberdeen AB25 2ZD, UK
Received 17 May 1996; revised 11 July 1996; accepted 16 July 1996

phase of 45% acetonitrile in 0.01 M phosphate buffer, pH 7, at a flow rate of 1 ml $min^{-1}$. Rhizoxin was detected as a single peak (retention time 4 min) by ultraviolet detection at 310 nm. The interassay coefficient of variation at 10 ng $ml^{-1}$ and 500 ng $ml^{-1}$ was <10%. The limit of detection was 1 ng $ml^{-1}$.

A population pharmacokinetic model was initially developed with data from the Glasgow phase I trial (Bissett *et al.*, 1992) and the current phase II population. The computer package NONMEM was used to estimate mean pharmacokinetic parameter values and their associated standard deviation in the population (Boeckman *et al.*). Estimates of the pharmacokinetic parameter values in the individual patient were obtained using patient plasma concentrations in a Bayesian algorithm (available in NONMEM).

The intersubject variability of the pharmacokinetic parameter values in the population was modelled using an exponential structure, i.e. $P_i = \bar{P}\ \exp\ (\eta_i)$, where $P_i$ represents the parameter (clearance, volume, etc.) estimate of the *i*th individual and $\bar{P}$ represents the typical value within the population. $\eta_i$ represents the individual difference between $P_i$ and $\bar{P}$ and $\eta$ is assumed to be normally distributed with mean zero and variance $\omega^2$.

The residual intrasubject variability was described using an additive and proportional model: $C_{ij} = \hat{C}_{ij}(1 + \varepsilon_{1ij}) + \varepsilon_{2jp}$ where $C_{ij}$ represents the *j*th measured concentration in the *i*th individual and $\hat{C}_{ij}$ is the *j*th predicted concentration in the *i*th individual. The differences in measured and predicted concentrations are represented by $\varepsilon_{1ij}$ and $\varepsilon_{2ij}$, which are assumed to be normally distributed with means zero and variances $\sigma_1^2$ and $\sigma_2^2$ respectively. Pharmacodynamic analysis was restricted to patients with evaluable rhizoxin pharmacokinetics. Rhizoxin AUC was calculated as: AUC = dose/systemic clearance. The median value of rhizoxin AUC for each grade of toxic or therapeutic effect was compared using the Kruskal–Wallis (K–W) test or the Mann–Whitney (M–W) test as appropriate. Groups were merged as indicated to provide sufficient numbers for valid analysis.

### Results

Blood sampling was performed in 69 of the 103 eligible patients enrolled in the four phase II rhizoxin studies (67%) (Kaplan *et al.*, 1996; Verweij *et al.*, 1996; Hanauske *et al.*, 1996). Of 18 centres involved in the phase II trials, 14 participated in the pharmacological evaluation of rhizoxin, representing seven countries throughout Europe. Blood sampling was performed in 78% of patients enrolled from these 14 centres (range 11–100%). Accrual of plasma samples varied by tumour type: NSCLC 24/29 (83%), melanoma 19/26 (73%), head and neck 18/31 (58%), and breast 8/17 (47%).

Rhizoxin was detectable in the plasma of 53 patients. The remaining patients were not evaluable because of an interfering plasma peak in the pretreatment sample (ten patients), no detectable plasma rhizoxin (five patients) and no early sampling secondary to acute toxicity (one patient). Rhizoxin was detectable in the plasma of 70% of patients at 10 min after infusion, 25% of patients at 20 min after infusion, and was detectable in only four patients at 30 min after infusion.

The time between sample collection and processing was unlikely to influence detection of rhizoxin, as little degradation (<10% of rhizoxin was observed *ex vivo* in human whole blood or plasma over 3 h.

Figure 1 shows that the rhizoxin plasma profile requires at least a two-compartment model to describe the data. When one- and two-compartment models were fitted to the data using NONMEM, the two-compartment model gave a better fit ($\chi^2 = 44$ with 2 d.f., $P < 0.001$). However, there were insufficient data to estimate reliably the values for all four parameters in the model (volume of distribution, elimination constant and intracompartmental constants). The distribution phase was very rapid and complete by 10 min after the end of the infusion, so only the 5 min after infusion sample gave information about distribution parameters. To overcome this problem, all measured concentrations up to and including 5 min after the end of the infusion was censored and a one-compartment model fitted to the remaining data from the seven phase I and 36 phase II patients. The parameter values and their associated errors estimated using this model are given in Table I.

Individual parameter estimates derived using the Bayesian algorithm implemented in NONMEM are summarised for all 36 phase II patients and for each tumour type in Table II. No significant correlation was observed between patient age and rhizoxin AUC.

The median AUC was not the same for all tumour types (Table II; K–W, $P = 0.03$). However, pairwise comparisons

**Figure 1** Concentration–time profile of rhizoxin in 37 phase II patients with more than one detectable plasma sample.

**Table I** Population parameter estimates from a one-compartment model NONMEM analysis

| | *Estimate* | *Standard error of estimate* |
|---|---|---|
| Clearance (l $min^{-1}$ $m^{-2}$) | 7.6 | 0.93 |
| $\omega^2$ clearance (% CV) | 0.375 (61%) | 0.146 |
| Volume of central compartment (l $m^{-2}$) | 128.0 | 41.4 |
| $\omega^2$ volume (% CV) | 0.395 (63%) | 0.293 |
| $\sigma_1^2$ (% CV) | 0.033 (18%) | 0.0153 |
| $\sigma_2^2$ (ng $ml^{-1}$) | 0.394 | 0.0347 |

**Table II** Median (and range) of individual parameter estimates for the 36 phase II patients and for each tumour type

| | *All patients* | *Breast* (n = *5*) | *Head and neck* (n = *9*) | *Melanoma* (n = *11*) | *NSCLC* (n = *11*) |
|---|---|---|---|---|---|
| Clearance (l $min^{-1}$ $m^{-2}$) | 8.4 (1.3–16.3) | 8.8 (1.4–14.5) | 10.8 (3.5–16.3) | 9.9 (6.4–16.0) | 6.4 (1.3–13.3) |
| Volume (l $m^{-2}$) | 122 (27–156) | 135 (28–156) | 103 (85–146) | 126 (81–135) | 122 (27–139) |
| Half-life (min) | 10.4 (3.5–19.2) | 10.5 (6.6–18.2) | 7.9 (3.6–19.2) | 9.2 (3.5–14.7) | 11.9 (6.8–17.2) |
| AUC (ng $ml^{-1}$ min) | 241 (115–1532) | 230 (139–1422) | 148 (115–414) | 203 (124–314) | 315 (150–1532) |

of the median AUC in each tumour type failed to identify significant differences when a Bonferroni correction was applied to account for multiple testing.

As predicted from the preclinical and phase I studies, toxicity from rhizoxin was primarily haematological (12% patients with grade III, 16% with grade IV neutropenia), gastrointestinal (5% patients with grade III stomatitis) and alopecia (complete in 23% of patients). In addition, pain at the tumour site was observed in five patients (Verweij *et al.*, 1996). No differences could be demonstrated in the median AUC at different toxicity grades for any of the measures of toxicity (Table III). No significant correlation could be demonstrated between the percentage reduction in WBC or the percentage in neutrophil count and rhizoxin AUC.

In the 36 patients evaluable for rhizoxin AUC, two patients achieved a partial response (PR), seven had stable disease (SD), and 23 had progressive disease (PD). Four patients were not evaluable in terms of tumour response. No complete responses were observed among the patients with evaluable rhizoxin pharmacokinetics. The nine cases with PR/SD (six NSCLC, two head and neck and one melanoma) had a significantly higher median AUC (314 ng ml$^{-1}$ min; range 138–1532) than the 23 patients with PD (222 ng ml$^{-1}$ min; range 115–1422) (Figure 2; M–W, $P=0.03$; 95% CI −16 to −264). The small number of patients with PR/SD prohibited separate statistical analysis for each tumour type.

**Figure 2** The relationship between rhizoxin AUC and tumour response (Mann–Whitney test, $P=0.03$). SD, stable disease; PR, partial response; PD, progressive disease. *Patients with very high rhizoxin AUC values.

**Figure 3** Theoretical relationship between rhizoxin AUC and measures of haematological toxicity (●, grade I/II; □, grade III/IV) and tumour response (○). Symbols represent data from the phase I (median 460 ng ml$^{-1}$ min) and phase II (median AUC 240 ng ml$^{-1}$ min) trials. The dashed lines for tumour response represent two possible outcomes from further escalation of systemic exposure: increased activity or plateau effect. There is currently not enough clinical data available to validate the *in vivo* presence of such relationships.

**Table III** Rhizoxin pharmacodynamics: comparison of median AUC and grade of toxicity

| *Toxicity* | | n | *Median AUC* | *Statistics* |
|---|---|---|---|---|
| Alopecia | Grade 0 | 7 | 252 ng ml$^{-1}$ min | K-W, $P=0.19$ |
| | Grade 1 | 20 | 187 | |
| | Grade 2 | 9 | 251 | |
| Asthenia | Grade 0 | 25 | 252 ng ml$^{-1}$ min | M-W, $P=0.94$; 95% CI -64 to 102 |
| | Grade 1–3 | 11 | 222 | |
| Pain | Grade 0 | 31 | 252 ng ml$^{-1}$ min | Insufficient data |
| | Grade 2–4 | 5 | 174 | |
| Skin | Grade 0 | 28 | 251 ng ml$^{-1}$ min | M-W, $P=0.98$; 95% CI -86 to 102 |
| | Grade 1–3 | 8 | 223 | |
| Stomatitis | Grade 0 | 22 | 253 ng ml$^{-1}$ min | M-W, $P=0.88$; 95% CI -58 to 102 |
| | Grade 1–3 | 14 | 223 | |
| WBC | Grade 0 | 13 | 273 ng ml$^{-1}$ min | K-W, $P=0.18$ |
| | Grade 1–2 | 14 | 204 | |
| | Grade 3–4 | 9 | 224 | |
| Neutrophils | Grade 0 | 16 | 252 ng ml$^{-1}$ min | M-W, $P=0.88$; 95% CI -70 to 102 |
| | Grade 1–4 | 20 | 227 | |
| Platelets | Grade 0 | 32 | 252 ng ml$^{-1}$ min | Insufficient data |
| | Grade 1–2 | 4 | 223 | |
| Haemoglobin | Grade 0 | 21 | 203 ng ml$^{-1}$ min | M-W, $P=0.68$; 95% CI -103 to 55 |
| | Grade 1–3 | 15 | 274 | |

M–W, Mann–Whitney test; K–W, Kruskal–Wallis test; CI, confidence interval for difference in median values.

### Discussion

Rhizoxin was rapidly eliminated from human plasma with a median clearance of 8.4 l $min^{-1}$ $m^{-2}$ and elimination half-life of 10.4 min. These results are consistent with those observed in phase I pharmacokinetic analysis in which the median clearance was 4.2 l $min^{-1}$ $m^{-2}$ and elimination half-life was 23.6 min. Rhizoxin was not detectable (<1 ng $ml^{-1}$) at 30 min after injection in 93% of plasma samples. A high degree of interpatient variability in rhizoxin disposition was observed in this study. Systemic clearance ranged from 1.3 to 16.3 l $min^{-1}$ $m^{-2}$ (CV=61%), while volume of distribution was 27–156 l $m^{-2}$ (CV=63%). A similar degree of variability was observed in the phase I trial (clearance, 2.0–4.6 l $min^{-1}$ $m^{-2}$; volume of distribution, 10.1–55.4 l $m^{-2}$) (Bissett *et al.*, 1992). In the current study, plasma rhizoxin concentrations measured 5 min after infusion varied from undetectable (<1 ng $ml^{-1}$) to 140 ng $ml^{-1}$ (median 10 ng $ml^{-1}$). Two patients had plasma rhizoxin concentrations (and AUC estimates) which are considerably higher than in other patients (Figure 2). Both patients were from the same centre, which raises questions as to the reason for the high concentrations. However, they have been included in the analysis. As pharmacokinetic studies were only performed with the first course of rhizoxin, direct measurement of intrapatient variability in rhizoxin pharmacokinetics was not possible.

The rapid elimination of rhizoxin makes pharmacokinetic analysis very difficult (McLeod *et al.*, 1996). Traditional methods of both compartmental and non-compartmental pharmacokinetic analysis require 2–3 blood samples for each variable to be estimated and data collection over at least three half-lives to allow confident calculation of pharmacokinetic parameters (Gibaldi and Perrier, 1982). Application of these 'rules' in the current study would have used 8–12 blood samples over ⩾30 min, beyond the point at which rhizoxin was detectable in patient plasma. The difficulties in obtaining accurate estimates of pharmacokinetic parameters for a two-compartment model were such that only a one-compartment model could be estimated. This required that early concentration information be censored to give estimates of post-distribution drug elimination.

Although unidentified species were not observed in the chromatograms from patient plasma samples, the production of metabolite(s) via rhizoxin metabolism may contribute to the rapid elimination from plasma. The systemic clearance was greater than liver blood flow (approximately 1.5 l $min^{-1}$) in all subjects, implying that extrahepatic metabolism or degradation was involved. Another potential mechanism of apparent clearance from plasma is binding to tubulin, rhizoxin's molecular target (Sullivan *et al.*, 1990). Several blood and tissue components, including platelets, are rich in tubulin (Wild *et al.*, 1995). While binding to platelet tubulin is a theoretical source of variability in rhizoxin disposition, *ex vivo* incubation in whole blood or plasma for up to 3 h had little influence on rhizoxin plasma concentrations.

Rhizoxin AUC was significantly higher in patients with NSCLC (Table II). The mechanism for this alteration is not clear. The patients in the NSCLC study did not have prior systemic chemotherapy, ruling out the influence of platinum complexes and related compounds on rhizoxin systemic clearance. As a considerable degree of overlap in systemic clearance was observed between the four tumour types, a larger number of patients would need to be evaluated to confirm that rhizoxin disposition is indeed different in NSCLC patients.

Although a high level of variability in the systemic exposure of rhizoxin was observed in this study, there was no evidence that rhizoxin AUC was related to drug toxicity. One contributing factor is the low degree of toxicity in this study. Even among frequently occurring toxicities, the majority of patients had grade I or II toxicity (Table III). Indeed, 44% of patients had no neutropenia. Another contributing factor is the absence of extensive measurement of haematological parameters, needed for accurate determination of nadir values. The lowest measured value was used to determine the % change in haematological parameters induced by rhizoxin therapy, which may differ from the actual nadir value. There is no easy solution to this commonly encountered difficulty for pharmacodynamic analysis, as daily analysis of the blood profile is not practical. Bayesian modelling techniques for estimation of individual patient neutrophil profile is in an early stage of development and may provide a useful approach in the future (Sonnichsen *et al.*, 1994).

Rhizoxin plasma AUC was statistically higher in patients achieving a PR or SD than those with progressive disease (median 314 *vs* 222 ng $ml^{-1}$ min, $P=0.03$). However, the response group contained nine patients, with the majority having stabilisation of disease only. The percentage of patients responding (PR+SD) was lower in those with pharmacological analysis than the whole study population (51% *vs* 28%) (Kaplan *et al.*, 1996; Verweij *et al.*, 1996; Hanauske *et al.*, 1996). Therefore, these results should be viewed with caution until confirmed by a larger clinical trial. Attention should also be paid to the choice of biological matrix for evaluating the therapeutic effect of highly potent drugs, such as rhizoxin. Variability in drug transport, intracellular processing and interaction with the cellular target are but a few of the influencing events between measurement of a drug in plasma and pharmacological activity. As measurement of drug concentrations or degree of inhibition in microtubule assembly in the tumour is not feasible for the majority of patients, use of an alternative biological end point may be in order (Cassidy and McLeod, 1995). Methods for determination of paclitaxel action on human platelet tubulin have been described following *in vitro* incubations and may be applicable for guiding the use of rhizoxin and other tubulin-interactive agents in future trials (Rowinsky *et al.*, 1988).

Another pharmacodynamic variable, which may be important for rhizoxin, is the amount of time that drug concentrations are maintained above a threshold concentration. This variable appears to be correlated with haematological toxicity in studies of other agents that interact with tubulin, such as paclitaxel (Gianni *et al.*, 1995; Huizing *et al.*, 1993). Relevant threshold concentrations are identified using individual patient pharmacokinetic parameters to simulate the concentration–time profile. In theory, this represents *in vivo* evidence of saturable biological processes, in which concentrations in excess of that required for binding the cellular target do not contribute to the pharmacological effect. Estimation of the time that rhizoxin plasma levels were above specific values was not determined in the current study, owing to the extensive assumptions in rhizoxin pharmacokinetic profile that would have to be made beyond 20 min after infusion. Future pharmacokinetic analysis using more sensitive assay methodology (gas chromatography with mass spectroscopy or enzyme-linked inmmunosorbent assay) will be required to explore the importance of threshold concentrations to rhizoxin toxicity and therapeutic activity.

The absence of a significant relationship between toxicity and rhizoxin systemic exposure in this study does not mean that one does not exist. The majority of patients in the pharmacological study (73%) had grade 0–II haematological toxicity after 2 mg $m^{-2}$, whereas only 7% of patients obtained an objective response. In the phase I study of rhizoxin, a dose of 2.6 mg $m^{-2}$ led to grade III/IV haematological toxicity in 87% of patients (Bissett *et al.*, 1992). These findings are consistent with the flat portion of the exposure–tumour response curve (e.g. Figure 3), where a relatively large increase in drug exposure does not translate into greater drug effect on the tumour. They also suggest that the *in vivo* concentration–effect curve for rhizoxin toxicity is very steep. Indeed, assuming *in vitro* principles of pharmacology hold true in humans, a concentration–effect curve, such as the theoretical example shown in Figure 3, may explain the clinical profile of rhizoxin. It is not known whether an increase in rhizoxin AUC will translate into greater anti-tumour activity or if a plateau in response will occur. Therefore, future trials using higher rhizoxin dosage, methods for reducing dose-limiting side-effects, and/or alternative dosage schedules should be considered.

In summary, rhizoxin is rapidly eliminated in humans, with rhizoxin detectable in the plasma of only 7% of patients by 30 min after injection. A large degree of interpatient variability was observed for all pharmacokinetic parameters. Rhizoxin plasma AUC was higher in patients achieving a therapeutic response than in those with progressive disease.

**Acknowledgements**

This study could not have been completed without the efforts of Ms te Velde and colleagues at the EORTC NDDO and the participating physicians, nurses, data managers and laboratory investigators of the EORTC ECSG and PAMM groups.

### References

BISSETT D, GRAHAM M, SETANOIANS A, CASSIDY J, KERR DJ, HENRAR R, MORRISON G AND KAYE SB. (1992). Phase 1 and pharmacokinetic study of rhizoxin. *Cancer Res.*, **52**, 2894–2898.

BOECKMAN AJ, SHEINER LB AND BEAL SL.(1992). *NONMEM IV User's Guide, Parts I–VII.* NONMEM Project Group, University of California: San Francisco, CA.

CASSIDY J AND MCLEOD HL. (1995). It is possible to design a logical development plan for an anti-cancer drug? *Pharmaceut. Med.*, **9**, 95–103.

GIANNI L, KEARNS CM, GIANI A, VIGANO L, LOCATELLI A, BONADONNA G AND EGORIN MJ. (1995). Nonlinear pharmacokinetics and metabolism of paclitaxel and its pharmacokinetic/pharmacodynamic relationship in humans. *J. Clin. Oncol.*, **13**, 180–190.

GIBALDI M AND PERRIER D. (1982). *Pharmacokinetics.* 2nd edn. Dekker: New York.

GRAHAM MA, BISSETT D, SETANOIANS A, HAMILTON T, KERR DJ, HENRAR R AND KAYE SB. (1992). Preclinical and phase I studies with rhizoxin to apply a pharmacokinetically guided dose escalation scheme. *J. Natl Cancer Inst.*, **84**, 494–500.

HANAUSKE AR, CATIMEL G, AAMDAL S, TEN BOKKEL HUININK W, PARIDAENS R, PAVLIDIS N, KAYE SB, TE VELDE A, WANDERS J AND VERWIEJ J FOR THE EORTC EARLY CLINICAL TRIALS GROUP. (1996). Phase II clinical trials with rhizoxin in breast cancer and melanoma. *Br. J. Cancer*, **73**, 397–399.

HENDRIKS HR, PLOWMAN J, BERGER DP, PAULL KD, FIEBIG HH, FODSTAD O, DREEF-VAN DER MEULEN HC, HENRAR REC, PINEDO HM AND SCHWARTSMANN G. (1992). Preclinical antitumour activity and animal toxicity studies of rhizoxin, a novel tubulin-interacting agent. *Ann. Oncol.*, **3**, 755–763.

HUIZING MT, KEUNG ACF, ROSING H, VANDER KUIJ V, TEN BOKKEL HUININK WW, MANDJES IM, DUBBELMAN AC, PINEDO HM AND BEIJNEN JH. (1993). Pharmacokinetics of paclitaxel and metabolites in a randomized comparative study in platinum-pretreated ovarian cancer patients. *J. Clin. Oncol.*, **11**, 2127–2135.

KAPLAN S, HANAUSKE AR, PAVLIDIS N, BRUNTSCH U, TE VELDE A, WANDERS J, HEINRICH B AND VERWEIJ J FOR THE EORTC EARLY CLINICAL TRIALS GROUP. (1996). Single agent activity of rhizoxin in non-small-cell lung cancer: a phase II trial of the EORTC Early Clinical Trials Group. *Br. J. Cancer*, **73**, 403–405.

MCLEOD HL, GRAHAM MA, AAMDAL S, SETANOIANS A, GROOT Y AND LUND B ON BEHALF OF THE EORTC EARLY CLINICAL TRIALS GROUP. (1996). Phase I pharmacokinetics and limited sampling strategies for the bioreductive alkylating drug EO9. *Eur. J. Cancer* **32**, 1518–1522.

ROWINSKY EK, DONEHOWER RC, JONES RJ AND TUCKER RW. (1988). Microtubule changes and cytotoxicity in leukemic cell lines treated with Taxol. *Cancer Res.*, **48**, 4093–4100.

SONNICHSEN DS, HURWITZ CA, PRATT CB, SHUSTER JJ AND RELLING MV. (1994). Saturable pharmacokinetics and paclitaxel pharmacodynamics in children with solid tumours. *J. Clin. Oncol.*, **12**, 532–538.

SULLIVAN AS, PRASAD V, ROACH MC, TAKAHASHI M, IWASAKI S AND LUDEUNA RF. (1990). Interaction of rhizoxin with bovine brain tubulin. *Cancer Res.*, **50**, 4277–4280.

TSURUO T, OH-HARA T, LIDA H, TSUKAGOSHI S, SATO Z, MATSUDA I, IWASAKI S, OKUDA S, SHIMIZU F, SASAGAWA K, FUKAMI M, FUKUDA K AND ARAKAWA M. (1986). Rhizoxin, a macrocyclic lactone antibiotic, as a new antitumour agent against human and murine tumour cells and their vincristine-resistant sublines. *Cancer Res.*, **46**, 381–385.

VERWEIJ J, WANDERS J, GIL TH, SCHOFFSKI P, CATIMEL G, TE VELDE A AND DE MULDER PHM FOR THE EORTC EARLY CLINICAL TRIALS GROUP. (1996). Phase II study of rhizoxin in squamous cell head and neck cancer. *Br. J. Cancer*, **73**, 400–402.

WILD MD, WALLE UK AND WALLE T. (1995). Extensive and saturable accumluation of paclitaxel by the human platelet. *Cancer Chemother. Pharmacol.*, **36**, 41–44.